# Revealing the Plastic Mode of Time-Dependent Deformation of a LiTaO_3_ Single Crystal by Nanoindentation

**DOI:** 10.3390/mi11090878

**Published:** 2020-09-21

**Authors:** Shengyun Zhou, Xianwei Huang, Congda Lu, Yunfeng Liu, Taihua Zhang, Yi Ma

**Affiliations:** 1 College of Mechanical Engineering, Zhejiang University of Technology, Hangzhou 310014, China; zhousy@yeefung.com (S.Z.); huangxw@zjut.edu.cn (X.H.); Liuyf76@zjut.edu.cn (Y.L.); 2 Key Laboratory E&M, Zhejiang University of Technology, Ministry of Education & Zhejiang Province, Hangzhou 310014, China; 3 Institute of Solid Mechanics, Beihang University, Beijing 100191, China; zhangth66@buaa.edu.cn

**Keywords:** lithium tantalite, nanoindentation, time-dependent deformation, crack, creep

## Abstract

Recently, instrumental nanoindentation has been widely applied to detect time-dependent plastic deformation or creep behavior in numerous materials, particularly thin films and heterogeneous materials. However, deformation mechanism at nanoindentation holding stage has not been well revealed hitherto. In the current work, nanoindentation holding tests with high loads were performed on a brittle LiTaO_3_ single crystal. The surface morphologies of residual impressions with various holding times were investigated. It was indicated that generation of secondary cracks and propagation of both main and secondary cracks were the dominating mechanism for time-dependent plastic deformation at the initial holding stage, and the density and length of cracks were invariable at the steady-state holding stage, which suggested a nonlocalized plastic deformation beneath the indenter. It could be concluded that time-dependent plastic deformation of brittle ceramic under nanoindentation is composed of instant cracking as the continuation of loading sequence and homogeneous creep flow by high shear-compression stress at room temperature.

## 1. Introduction

Creep is classically defined as a time-dependent plastic deformation, which is occurred during elastic holding and facilitated by high temperature [1]. Creep resistance is essentially important to engineering materials which bear long-term elastic stress and high-temperature structures with active atomic mobility. Over the last century, evaluation of creep behavior has been on the cutting edge of mechanical characterization from both engineering requirement and scientific interests [2,3]. The testing requirements of conventional creep measurement (according to ASTM standards) are rigorous [4]. The large standard size of specimen and long-time (thousands of hours) duration are necessary. This greatly hinders the exploration of creep behavior and mechanism in those brittle, small-sized and heterogeneous materials. In view of limitations of conventional uniaxial holding test, nanoindentaton technology has been widely used to detect creep deformation at the nano/micro scales in recent years [5,6,7,8,9]. Due to its ultrahigh testing accuracy, it is much more time-saving to obtain creep flow by nanoindentation than conventional methods. Furthermore, the effects of loading rate, indentation length scale and holding strain on creep behavior could be conveniently investigated relying on instrumental nanoindentation. Particularly, nanoindentation creep measurement has been performed in thin films and nanopillars [10,11,12,13], and creep features of the new-structure materials such as nanocrystalline alloys, metallic glasses and high-entropy alloys, etc. were revealed [14,15,16,17,18,19].

To weaken thermal drift influence on the mechanical response, nanoindentation load-holding tests were commonly conducted at room temperature. At the onset of the nanoindentation holding stage, plastic deformation was generally occurred particularly by adopting Berkovich indenter. In this scenario, the mechanism of time-dependent plastic deformation under nanoindentation could be much different to that of the thermally activated creep flow by conventional method. To the authors’ best knowledge, the nanoindentation creep mechanism was not well revealed and merely qualitatively discussed from the viewpoint of high-temperature creep [3]. It is much more difficult to directly observe the changes of plastic morphology during holding stage at the microscale by either in-situ or ex-situ detections. Provided that creep flow occurs at the plastic zone suffered maximum shear stress beneath indenter, the cross-section of residual impression needs to be probed by scanning electron microscope (SEM) or transmission electron microscopy (TEM). In the meanwhile, new structure damage could be induced by focused ion beam (FIB) cutting, which would greatly disturb the acquisition of creep mechanisms. Furthermore, the plastic mechanisms, i.e., dislocations, twins and microcracks could stochastically appear due to the nonuniform distribution of defects at the nano- or microscale [20]. As a consequence, the change of plastic morphology induced by creep deformation could be very difficult to discern at different locations.

Lithium tantalite (LiTaO_3_) has been extensively used in the functional devices like laser, communication, acoustic fields as an excellent piezoelectric material [21]. In order to obtain better functional performance, smooth surface and ultrathin thickness of LiTaO_3_ wafer are required. Due to its soft-brittle nature, catastrophic fracture of LiTaO_3_ is always occurred during precise machining [22,23]. As the surface of LiTaO_3_ wafer suffers long-term point-contact deformation during grinding and polishing, nanoindentation exploration has attracted lots of attentions on revealing the mechanical properties and deformation features of LiTaO_3_ at the microscale [24,25,26,27]. In the authors’ previous work, room-temperature creep deformation was reported in this high-melting-point ceramic [28,29], and cracking is the dominating deformation mode under high-load nanoindentation for brittle solids, which could be directly detected from the surface morphology of residual impression. Relying on nanoindentation technology, researchers investigated the fracture features and estimated the fracture toughness in a lot of brittle ceramics and their composites [30,31,32]. In this work, we intend to unfold the mechanism of time-dependent plastic deformation in brittle ceramic via the evolution of surface cracks after holding stage. The deformation features at different creep stages, i.e., instantaneous and steady-state segments were studied according to the changes of size and density of cracks. Furthermore, grid nanoindentations were performed to eliminate the local occasionality of crack morphology for each holding condition.

## 2. Materials and Methods

**Figure 1:**
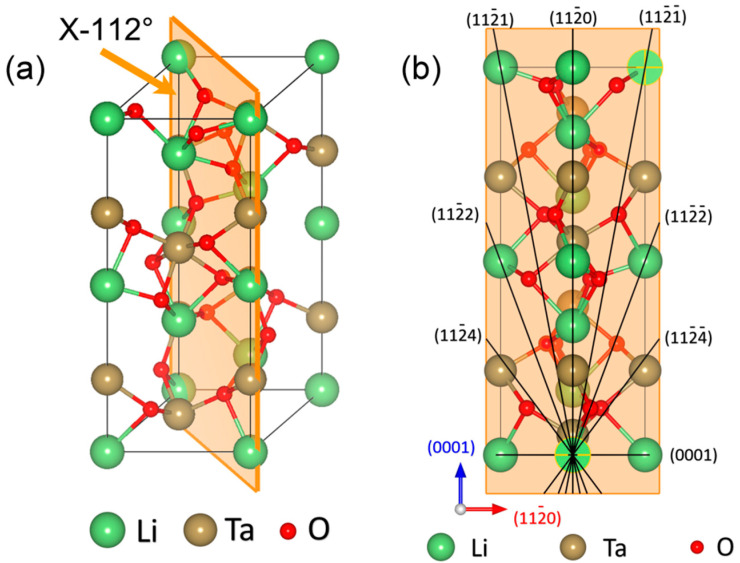
(**a**) Schematic illustration of atomic arrangements of LiTaO_3_ single crystal, the X-112° plane ($\bar{1}$102) is depicted. (**b**) Slip system perpendicular to the X-112° plane.

**Figure 2:**
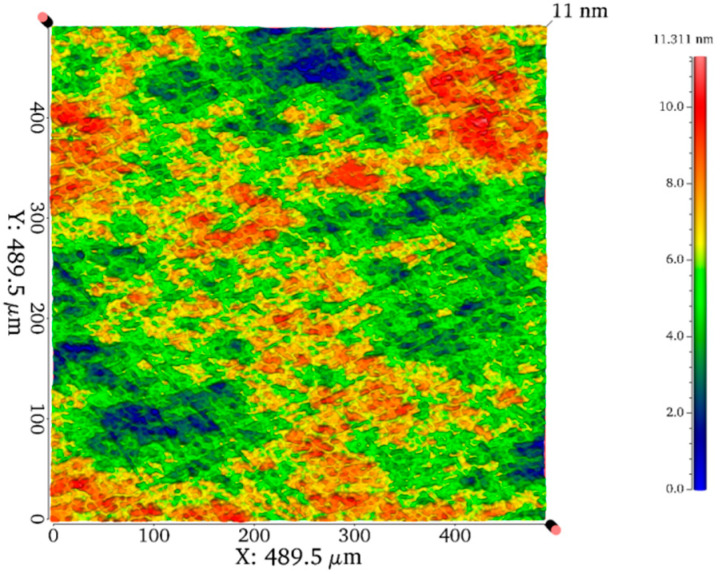
Top view of the surface morphology after precise polishing process.

A LiTaO_3_ single crystal belongs to the trigonal R3c space group with ion bonding structure and is commonly depicted by a hexagonal axis. Figure 1a shows the schematic illustration of the atomic arrangement of a LiTaO_3_ single crystal and the typical orientation ($\bar{1}$102) also known as X-112° plane. The slip system perpendicular to X-112° plane was exhibited in Figure 1b. Prior to nanoindentation, the X-112° plane was carefully polished to a mirror surface, of which roughness *R*_a_ was lower than 1 nm. Diamond abrasive at the average size of 2 um (8000#) and polyurethane polish pad were adopted on the Nanopoli-100. After 45-min polishing, LiTaO_3_ samples were carefully cleaned in the anhydrous alcohol by ultrasonic cleaning [33]. Top view of surface morphology was observed by an optical 3D surface profilometer (Talysurf i-Series) on the area of 500 × 500 μm^2^ as exhibited in Figure 2, by which the surface roughness *R*_a_ could be measured.

Nanoindentation load-holding tests were performed on Agilent Nano Indenter G200 at ambient temperature of 23 °C by air condition. A spherical tip with nominal radius of 5 μm was adopted to study the crack evolution during nanoindentation holding. The effective spherical tip radius was obtained as 2.95 μm by calibrating on the standard fused silica. The loading rate and peak load were fixed to 2 mN/s and 150 mN, respectively. Under the current testing conditions, both the residual impression and crack feature at the beginning of duration could be clearly captured by scanning electron microscope (SEM). Four individual holding segments at peak load were adopted to investigate the time-dependent plastic deformation under nanoindentation, which were ranged from 0 to 1500 s (0, 250, 500 and 1500 s). It should be pointed that nanoindentation holding test was performed individually, rather than cyclic loading at the same location. Sixteen independent measurements (a 4 × 4 matrix with 50 μm interval between two adjacent locations) were conducted for each case. All the holding tests were launched until thermal drift reduced to 0.03 nm/s. Meanwhile, thermal drift (*n*, generally within the range from −0.05 and +0.05) correction which was calibrated at 10% of the maximum load during the unloading process was strictly performed for each test. The reduced nanoindentation displacement could be obtained by *h*-*t × n*. The morphologies of all the residual impressions were observed by SEM (sigma hv-01-43). All the sixteen residual impressions at each holding condition were carefully detected, and the count and size of cracks were recorded.

## 3. Results and Discussion

**Figure 3:**
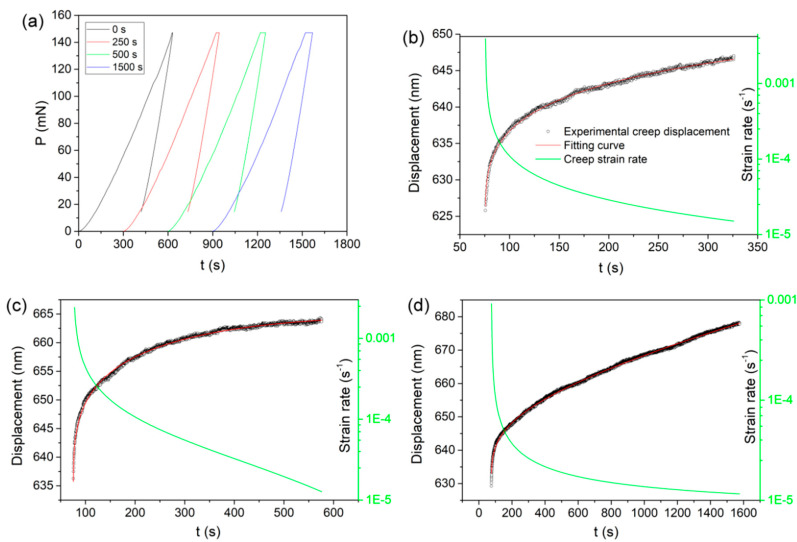
(**a**) Typical *P*-*h* curves of load-holding tests with various duration and the time-dependent plastic deformations were plotted as a function of holding time (**b**) 250 s, (**c**) 500 s and (**d**) 1500 s. An empirical law was adopted to fit the creep curves and the corresponding strain rate could be deduced. Both creep fitting line and strain rate were plotted as a function of holding time.

Figure 3a exhibits the representative load versus displacement (*P*-*h*) curves for the holding tests with different durations. Clearly, the permanent deformation continuously appeared at the holding stage, and the time-dependent plastic deformation was more pronounced with increasing holding time. The *P*-*h* curves exhibited a well repeatability for each holding time. Figure 3b–d show the representative pressed displacements or creep curves at the three holding stages, which were plotted as a function of holding time. The time-dependent plastic deformation exhibited a typical feature of nanoindentation creep flow, in which displacement was quickly increased at the beginning and then turned to be slowly and almost linearly increased with holding time, and the nanoindentation deformation during holding stage exhibited a well repeatability that the typical creep flows were almost overlapped for each case as shown in Figure S1 of Supplementary Material. The creep curves can be well fitted (R^2^ > 0.99) by an empirical law [34]:

*h*(*t*) = *h*_0_ + *a*(*t* − *t*_0_)^*b*^ + *kt* (1)

where *h*_0_, *t*_0_ are the displacement and time at the beginning of holding stage, *a*, *b*, *k* are the fitting constants. Based on the creep fitting lines, the nanoindentation strain rates of time-dependent plastic deformation under spherical tip were estimated by $\dot{\varepsilon }=\frac{1}{\sqrt{A}}\frac{d\sqrt{A}}{dt}$ where *A* is the contact area, equal to 2*πRh_c_*. *R* is the tip radius, the contact depth *h_c_* could be deduced as *h_c_* = *h* − *ε* × *P*/*S*, where *h* is the recorded indenter displacement, *ε* = 0.75 for a spherical tip, *S* is the contact stiffness [35]. It should be mentioned that the contact stiffness is almost linearly increased with pressed depth. In the current work, the displacement enhancement during holding stage could be less than 10% of the initial holding depth. Hence we simply adopted the stiffness at the unloading segment for each test to estimate the strain rate during the whole duration. The strain rates during holding stage were plotted as a function of holding time and also shown in Figure 3b–d. At the very beginning of the holding stage, the strain rate could be approached to the order of magnitude of 10^−3^ s^−1^ (the difference of strain rate among the three holding stages was due to the imperfection of the fitting lines at the initial 1~2 s) and then decreased to abut 1 × 10^−4^ s^−1^ as holding time increased to about 50~100 s. At the ending of each holding stage, the average strain rates were about 1.8 × 10^−5^, 1.45 × 10^−5^ and 1.15 × 10^−5^ s^−1^ for the 250, 500 and 1500 s holdings, respectively. This means the time-dependent plastic deformation turned into the steady state from the 250 holding as the strain rate was very slowly changed. The strain rate at the onset of holding stage could be obtained by ${\dot{\varepsilon }}_{i}=\frac{\dot{P}}{2P}$ ($\dot{P}=\frac{dP}{dt}=\frac{dAH}{dt}=\frac{2HA}{\sqrt{A}}\frac{d\sqrt{A}}{dt}=2P\dot{\varepsilon }$, *H* is hardness) [36], where $\dot{P}$ was the constant loading rate equal to 2 mN/s and *P* was the peak load 150 mN. Thus the strain rate of initial time-dependent plastic deformation was comparable to that (0.0067 s^−1^) of loading sequence.

**Figure 4:**
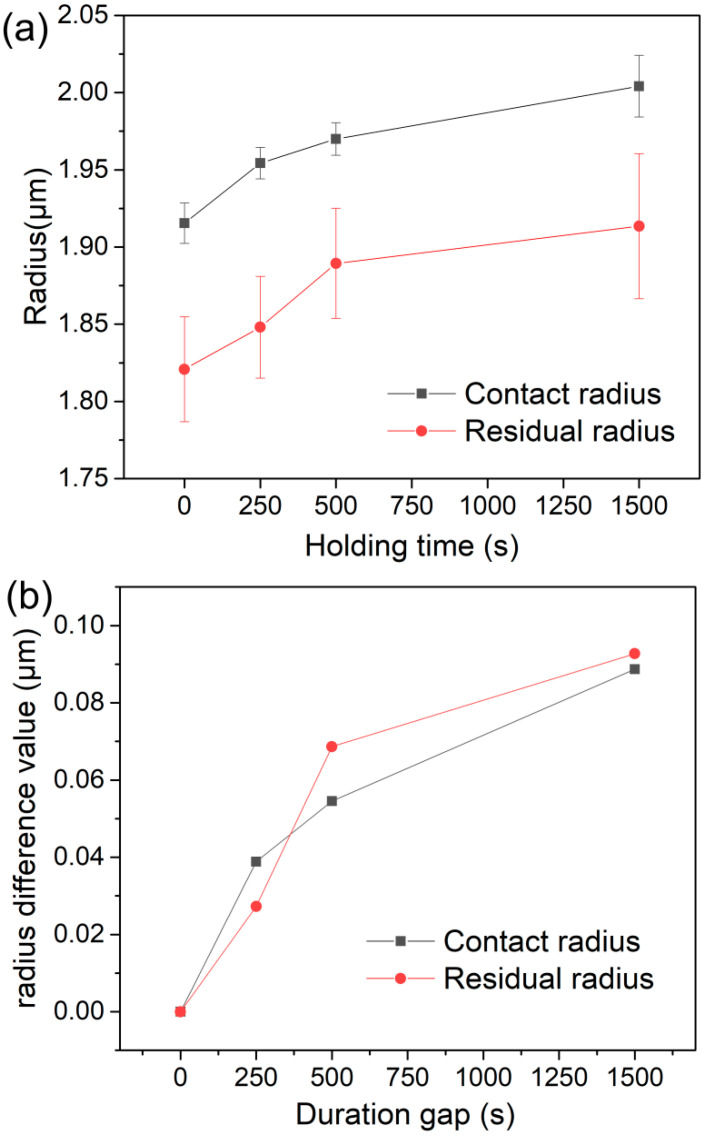
(**a**) The residual radii measured from scanning electron microscope (SEM) observation and the contact radii at the end of holding stage, which were plotted with holding time. (**b**) The increased values of contact and residual radii after holding stage.

The increased displacements Δ*h* at the end of holding stage were recorded for each measurement, the mean values were 20, 33.5 and 56.6 nm for the 250, 500 and 1500 s holdings, respectively. The mean contact radii at the peak load could be obtained by $a=\sqrt{2\pi Rh_{c}}$ in which $h_{c}$ is the contact depth equal to $h-0.75\frac{P}{S}$, *P* is the peak load and *S* is the contact stiffness. As shown in Figure 4a, the mean contact radii were 1.92, 1.95, 1.97 and 2 μm for instantaneous loading and three holding stages. Accordingly, the increased contact radius after holding stage could be estimated by $∆a=a-a_{0}$, as exhibited in Figure 4b.

**Figure 5:**
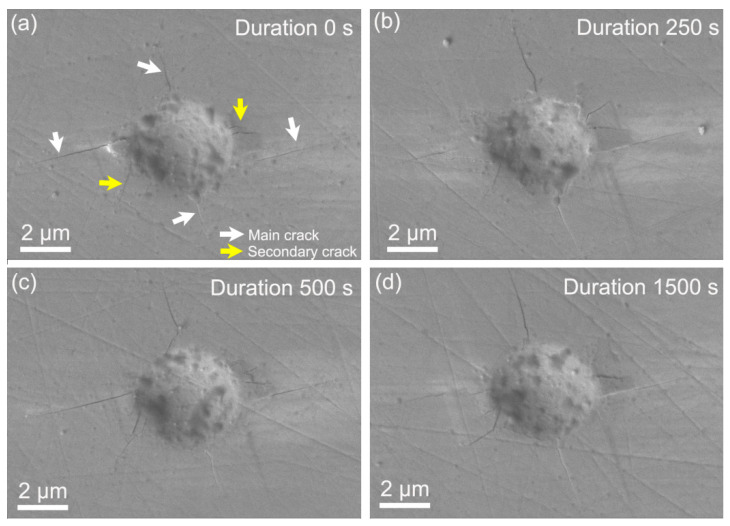
The surface morphologies of residual nanoindentation impressions by SEM observation on the samples at the holding stages of (**a**) 0 s, (**b**) 250 s, (**c**) 500 s and (**d**) 1500 s.

Figure 5 shows the typical surface morphologies of residual impressions at different holding times by SEM. Clearly, the microcrack was the dominating deformation manner of LiTaO_3_ under spherical nanoindentation. The radii of residual impressions were carefully measured for all the tests as also exhibited in Figure 4a. In compared to contact radius at peak load, the residual radius was evidently smaller due to the elastic recovery after unloading. Obviously, residual radius was enlarged with holding time, which substantially confirmed the occurrence of time-dependent plastic deformation under nanoindentation. Furthermore, the enlarged values of both contact and residual radii were very close for each holding stage, as exhibited in Figure 4b.

**Figure 6:**
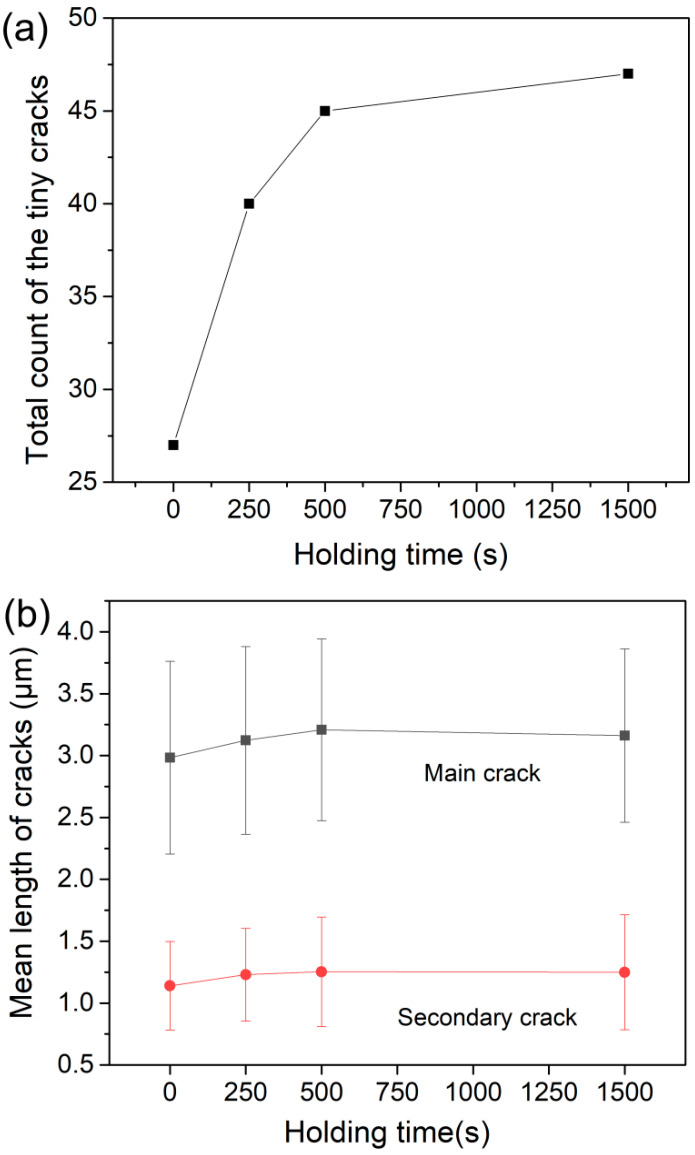
(**a**) Total count of secondary cracks; (**b**) mean lengths of the main and secondary cracks under different holding conditions.

**Figure 7:**
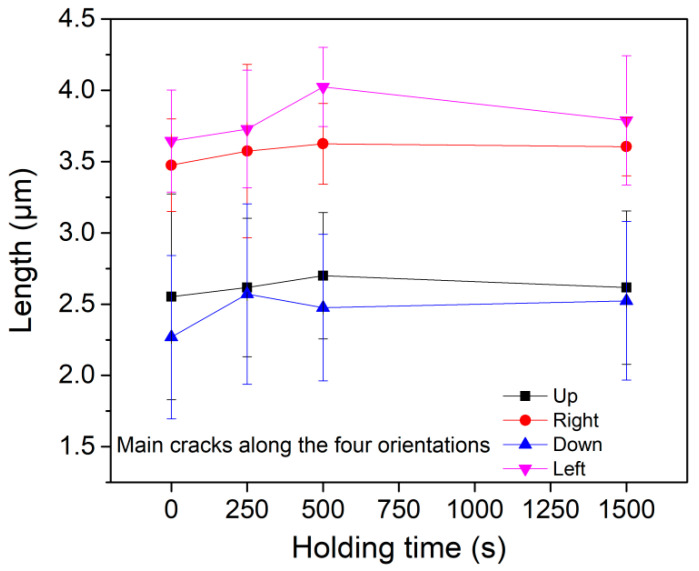
The correlation between main crack length and holding time along the four directions.

From the SEM images, it could be seen that symmetric cracks of several micrometers in length appeared along four directions with an angle of 90 degrees, as indicated by white arrows in Figure 5a. Here, we defined these primary cracks as the main crack. According to the slip system perpendicular to X-112° in Figure 1b, the ($\bar{1}$120) and (0001) might be the slip planes during nanoindentation deformation. Besides, several tiny cracks could also be observed, of which orientations were disordered as indicated by yellow arrows. The four main cracks appeared in all the residual impressions, thus its total count was constant. As exhibited in Figure 6a, the total count of secondary cracks in sixteen tests was dramatically increased from 27 to 40 after 250 s holding, and then secondary cracks were slowly increased from 40 to 47 by increasing holding time to 1500 s. Figure 6b shows the mean lengths of main and secondary cracks, which were both increased about 100 nm after 250 s holding and nearly invariable by further increasing holding time. In addition, mean lengths of the main cracks along four orientations were summarized in Figure 7. All the main cracks followed a similar variation trend with increasing holding time as shown in Figure 6b, which further illustrated a uniform extension of cracks during holding stages. According to statistical results in Figure 6, the main mechanisms of time-dependent plastic deformation in LiTaO_3_ on the three holding stages could be qualitatively unfolded, relying on the length evolution of pre-existing main cracks and generation of new secondary cracks.

For the initial holding stage (0~250 s), the total count of secondary cracks quickly increased from 27 to 40 in a total of sixteen tests. Meanwhile, the mean lengths of both main and secondary cracks were enlarged ~100 nm simultaneously, which held at approximately 3.15 and 1.25 μm, respectively. We can conclude that the initial time-dependent plastic deformation was induced by both generation of secondary cracks and elongation of main and secondary cracks. Consequently, deformation strain rate at this stage was evidently higher than the subsequent stage and comparable to the strain rate at instant deformation at the same load. The deformation manner and strain rate at initial holding stage could even be regarded as the continuation of loading sequence by stress overshoot [37].

As related to deformation behavior on the second holding stage, i.e., 250~500 s, the displacement was almost linearly increased with holding time, which represented a typical steady-state creep flow. In this holding stage, the strain rate was gradually decreased from about 3 × 10^−5^ to 1 × 10^−5^ s^−1^, as indicated in Figure 3. However, plastic deformation on this stage could not be negligible given that both contact and residual radii were evidently increased as exhibited in Figure 4b. In this case, the localized crack evolution was greatly depressed whilst still discernable that five secondary cracks were increased at the end of 500 s holding and the mean length of main cracks were weakly increased. In view of the total sixteen measurements, such low increase of secondary cracks could be insignificant and even attributed to the testing error by detecting at different regions. It should be pointed that the main cracks were clearly extended during this stage from results in Figure 6b and Figure 7. Accordingly, we conceived that this stage could be the interim period, in which the local cracking deformation was depressed or even disappeared and homogeneous creep flow were occurred.

For the time-dependent deformation on third holding stage (500~1500 s), its strain rate was slowly reduced to less than 2 × 10^−5^ s^−1^, and the slow increase of residual radius also confirmed the sluggish plastic deformation in this stage. Obviously, the count and length of main and secondary cracks were invariable on the whole stage. In this scene, the time-dependent plastic deformation could be drove by homogeneous creep flow, notwithstanding its mechanism should be distinct to high-temperature creep deformation. The nanoindentaiton strain rate could be transformed to uniaxial strain rate empirically by $\varepsilon_{u}=0.09\varepsilon_{I}$ [38]. Thus the equivalent strain rate at this stage was at the magnitude of 1 × 10^−6^ s^−1^ order. From the perspective of deformation kinetics, plastic deformation under such low strain rate was already beyond the scope of quasistatic deformation, and entirely met the requirement of creep deformation. In comparison, the herein local creep strain rate under high shear stress were within the strain rate ranges by macroscopical creep tests, i.e., bending, compressure and tension at high temperature and low stress. In Duong’s report, the steady-state creep strain rate of a single crystal calcium oxide was in the range approximately from 10^−7^ to 10^−5^ s^−1^ at 1350 °C under low stress (5~15 MPa) [39]. Lee studied the creep flow of alumina and silicon nitride at 1000 °C, the creep strain rates increased from 10^−^^6^ to 10^−^^4^ s^−1^ by enhancing the applied stress from 20 to 100 MPa [40]. In Jing’s work, the creep strain rate changed from 10^−8^ to 10^−5^ s^−1^ for SiC/SiC composites by increasing holding stress [41]. For the single crystal LiTaO_3_, of which melting point was higher than 1600 °C, its room-temperature creep deformation or homogeneous plastic flow was rarely studied, as well as its high-temperature creep behavior. The occurrence of creep deformation and its mechanism should be discussed.

At the onset of the holding stage, the peak load (150 mN) had already exceeded the nanoindentation yielding point of LiTaO_3_ single crystal [27]. The plastic region suffered high shear-compression stress beneath spherical tip, which was considerable. A “quasiplastic” deformation could be assumed under nanoindentation due to the nonuniform stress and constraint effect by elastic surroundings even for brittle materials [42]. Accordingly, catastrophic cracking was unable to immediately occur in LiTaO_3_ even though the deformation strain exceeded its elastic limit by nanoindentation. Therefore, the present main cracks along symmetrical directions were induced by high-stress squeezing and the disordered secondary cracks were formed from the twinning deformation at those “fertile” places with defects [43]. In compared to conventional creep test by uniaxial elastic holding, the stress and strain herein were much higher and structure state was severely agitated beneath the spherical indenter. Notwithstanding that holding tests were performed at room temperature, the high stress and severely plastic deformation at the onset of holding stage could facilitate the occurrence of creep mechanisms such as dislocation and grain boundary climb/glide (atomic diffusion was not considered at room temperature) [3]. As the maximum stress appeared right below (~*a*/2) the indenter, the time-dependent plastic deformation at the steady-state stage was expected to occur beneath indenter. Thus the little change of cracks morphology on the surface as holding time increased from 250 to 1500 s could be understandable.

In brief, the deformation mechanisms herein during the holding stage could be generally determined as crack evolution at transient stage and homogeneous creep flow at the steady-state stage, respectively. In the interim, segment between the two stages, crack extension and creep flow could occur simultaneously. It should be noted that the crack-dominated holding stage was transient and could be expected approximate 0~100 s, based on the change of strain rate at holding stage. Besides, the critical point for the pure creep deformation could be much earlier than 500 s. According to the changes of strain rate and deformation morphology, the mechanisms of time-dependent plastic deformation could be qualitatively discerned, while the specific transition time for different deformation manners was difficult to trace. Basically, the transition of deformation mode would largely rely on the testing conditions, e.g., crack-dominated deformation segment could be increased by increasing the applied stress or strain and strain rate of the loading sequence. For the homogeneous creep flow in brittle LiTaO_3_ single crystal, it was rarely studied and its mechanism was unknown under both uniaxial and nanoindentation holdings. In comparison to metals and alloys, it requires much more experiments and simulations in future to reveal the creep behavior and mechanism of LiTaO_3_, and we simply discussed the room-temperature creep deformation in LiTaO_3_ from the view of creep mechanisms of alloys. In the current work, we reported that the transient stage of time-dependent plastic deformation was crack-dominated rather than homogeneous creep flow in brittle ceramic, for the first time. It is promising that localized plastic deformation could play an important role at the nanoindentation holding stage for other materials provided holding time is limited. Importantly, it suggests that the determinations of creep behavior including creep strain and strain rate sensitivity by nanoindentation could be misleading at short duration.

## 4. Conclusions

In summary, the time-dependent plastic deformation of a LiTaO_3_ single crystal was investigated relying on a nanoindentation constant load-holding method. The permanent plastic deformation clearly occurred whilst its strain rate was precipitously reduced with increasing holding time. The density and length of cracks around the residual impression were evidently increased after 250 s holding. By further extending holding time, the morphology of surface cracks was rarely changed. Two distinct deformation mechanisms were informed for the time-dependent plastic deformation in this brittle ceramic under high shear-compression stress. At the beginning of the holding stage, generation of secondary cracks and propagation of both main and secondary cracks were the dominating deformation manners, which was consistent to high strain rate of this stage. At the steady-state segment of holding stage, of which strain rate was approximately lower than 10^−5^ s^−1^ and very slowly changed with holding time, the time-dependent plastic deformation could be ascribed to homogeneous creep flow which occurred in the severely plastic region beneath indenter. Furthermore, there existed an interim stage between the local cracking and homogeneous creep deformations, where there was deformation by cracking extension and creep flow simultaneously.

## Supplementary Materials

The following are available online at https://www.mdpi.com/2072-666X/11/9/878/s1, Figure S1: (a) Typical *P*-*h* curves of load-holding tests with various duration and the time-dependent plastic deformations were plotted as a function of holding time (b) 250 s, (c) 500 s and (d) 1500 s.
