# Supplementary material for: Revealing the Plastic Mode of Time-Dependent Deformation of a LiTaO3 Single Crystal by Nanoindentation
Source: Micromachines (Basel). 2020 Sep 21;11(9):878. doi: 10.3390/mi11090878 (PMC7569801; doi:10.3390/mi11090878)
Supplement: Supplementary file 1 [file micromachines-11-00878-s001.pdf]

Supplementary Materials: Revealing the Plastic Mode of Time-Dependent Deformation of a $LiTaO_3$ Single Crystal by Nanoindentation

Shengyun Zhou, Xianwei Huang, Congda Lu, Yunfeng Liu, Taihua Zhang and Yi Ma

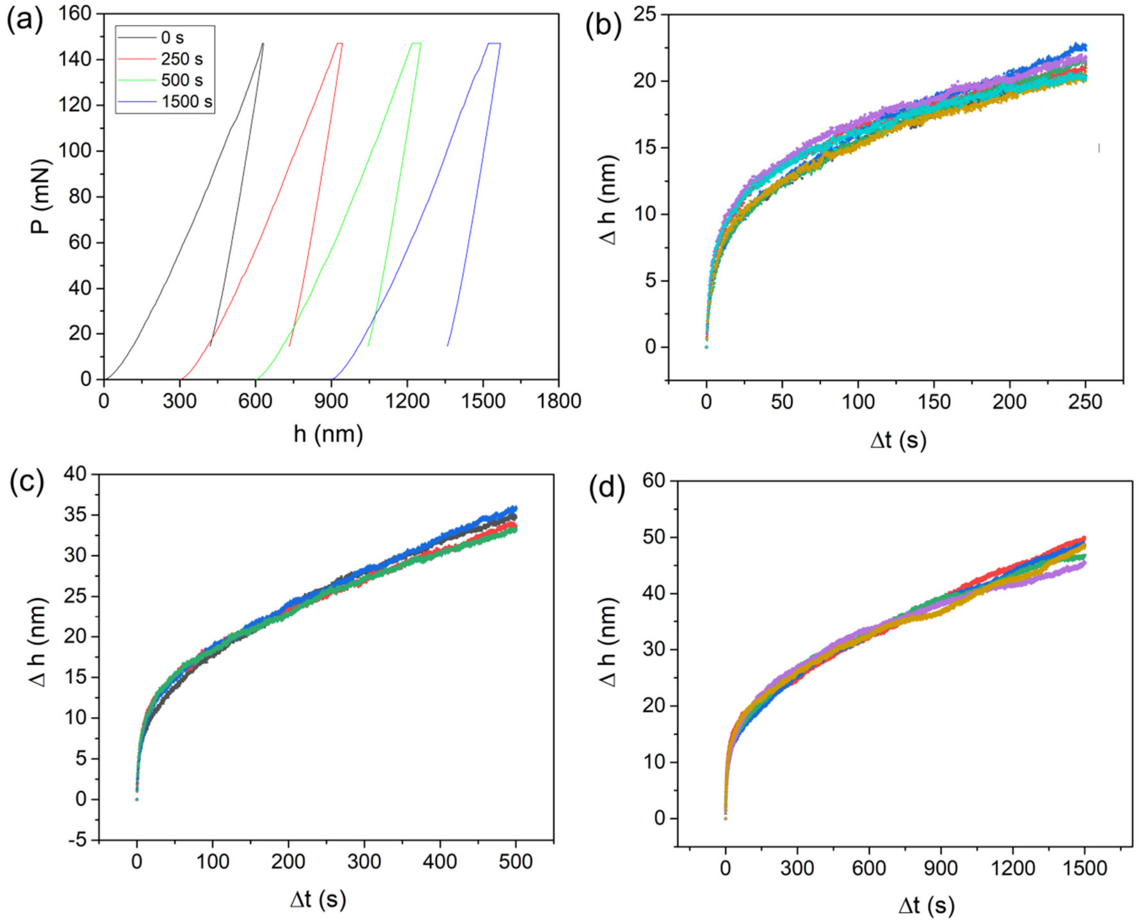

Figure S1. (**a**) Typical *P-h* curves of load-holding tests with various duration and the time-dependent plastic deformations were plotted as a function of holding time (**b**) 250 s, (**c**) 500 s and (**d**) 1500 s.
